# What do nurses experience in communication when assisting in robotic surgery: an integrative literature review

**DOI:** 10.1007/s11701-024-01830-z

**Published:** 2024-01-27

**Authors:** Lian Lee, Kathleen Greenway, Sue Schutz

**Affiliations:** Present Address: Oxford Brookes University, Oxford, UK

**Keywords:** Robotics, Robotic-Assisted Surgery, Perioperative Nursing, Nurses, Communication

## Abstract

**Background:**

Communication in surgery is integral to the fundamentals of perioperative nursing practice and patient safety. Research exploring team communication in robotic-assisted surgery (RAS) is evident in the literature but little attention has been focused on how the experiences of operating room nurses' communication affect safety, practice and patient care outcomes.

**Objective:**

To synthesise current evidence regarding communication during robotic-assisted surgery as experienced by registered nurses.

**Design:**

An integrative literature review informed by Whittemore and Knafl's (2005) methodology was used to conduct a rigorous analysis and synthesis of evidence.

**Methods:**

A comprehensive database search was conducted using PRISMA guidelines. CINAHL, Pubmed, PsychINFO and British Nursing Web of Science databases were searched using a Boolean strategy.

**Results:**

Twenty-five relevant papers were included in this literature review. Thematic analysis revealed two main themes with four related subthemes. The two main themes are: ‘Adaptive operating room nursing in RAS’ and ‘RAS alters team dynamics’. The four subthemes are: ‘Navigating disruptions in RAS’, ‘RAS heightens interdependence on team working’, ‘Augmented communicative workflow in RAS’, and ‘Professional empowerment to speak up’.

**Conclusions:**

This integrative review identifies how current research largely focuses on communication in the wider OR team. However, current evidence lacks the input of nurses. Therefore, further evidence is needed to explore nurses' experiences to highlight their perspectives.

**Clinical Relevance:**

Robotics significantly benefit patients, and this review identifies different challenges that robotic-assisted surgery nurses encounter. A better understanding of the communication from the perspective of nurses is needed to guide future research, practice education, policy development and leadership/management.

**Supplementary Information:**

The online version contains supplementary material available at 10.1007/s11701-024-01830-z.

## Introduction

Since the first robotic-assisted surgery (RAS) in the 1980s [18], surgical robotics has evolved rapidly in the healthcare industry and in research globally [19, 20]. The Association of British HealthTech Industry's (ABHI) [1] on *New Models of Surgical Care* recognises RAS as a strategy for driving the cancer backlog by influencing rapid updates of robotic surgery amongst surgical colleagues. Robotics is no longer only servicing elective surgery, and there has been growing surgical interest since Covid-19 pandemic and in emergency robotic-assisted surgery [28, 37]. The operating room (OR) is a complex environment and introducing new technology brings new challenges to perioperative teams and practice. Robotic surgery differs from conventional surgery, because the surgeon sits at the console away from the operating table, unlike traditional surgery where the surgeon directly handles the surgical instruments at the bedside. In RAS, the surgeons are being supported by the robotic system with its precision tools and powerful technology to guide the operation away from the bedside [20]. The Institute of Medicine report in [22], *'To Err is Human: Building a safer health system'* emphasised communication as the key means of improving safety and efficiency in surgery. The focus on intraoperative communication and interaction is a critical subject in healthcare practice as robotic surgery advances [4, 12, 29, 31, 34, 38, 43]. The surgeons are now hyperconnected with newest digital and virtual technologies, and remote surgery and tele-robotics surgery are no longer fiction but a reality as reported in medias and discussed in surgical conferences. In addition, the significant initiative in enabling Artificial Intellegence (AI) integration in robotics surgery as measures to enhance surgical training with a multidisciplinary collaboration amongst the surgeons, engineers, and software developers will bring a new generation of autonomous surgery in the simulation and practice setting [2, 33]. Therefore, the perioperative nursing practice ought to be reviewed as the surgeons are getting more digitally connected and the imminent of an AI integration in the advanced surgical tools. Ultimately, the human elements of perioperative practice and patient care remain central to every surgical innovation despite advance precision surgery that will improve the surgical outcomes. The fundamentals of perioperative nursing care emphasise the part communication plays in patient safety [3, 6], which will be pivotal to implementation of advanced precision surgery. Despite an increased focus on Human Factors and patient safety in RAS [12, 29, 35, 36], there remains little emphasis on nurses' communication experiences when assisting in RAS.

## Methods

**Fig. 1:**
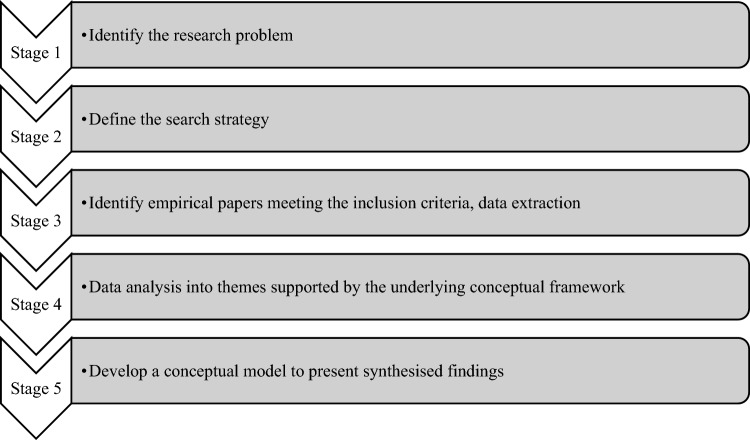
Stages of the integrative review using Whittemore and Knafl's [48] five-stage method

An integrative approach, informed by Whittemore and Knafl’s [48] methodology, was used to structure a rigorous analysis and synthesis of data for this review (Fig. 1). A mix of studies with various methodologies was included in this review (Whittemore et al., 49). Literature included in this review met clear and specific criteria to collect data in an unbiased manner, especially important in an area lacking empirical sources [48].

### Search strategy

A comprehensive database search was conducted using PRISMA guidelines to promote transparency in the search process [32]. This review sought to understand the communication experienced by the nurses in RAS and to determine how much has been explored in peer-reviewed publications. Hence, the search terms were broad to capture a wide variety of data relevant to the aims. The Cumulative Index to Nursing and Allied Health Literature (CINAHL), PubMed, PsychINFO and British Nursing Web of Science databases were searched using a Boolean strategy. Further literature searches included reference lists of previous literature reviews. An alert was set up on all databases to avoid any missing publications while undertaking the review. Data were extracted manually, organised, and analysed using Whittemore and Knafl’s [48] 5 stages method (Fig. 1). Any papers of uncertain eligibility were discussed with the authors^2,3^, to reach a consensus. Search terms were developed using keywords/synonyms from the review aim. A Boolean strategy was used using keywords (Table 1) based on database specifications, including reference to search terms using MeSH headings (Table 2). Synonyms were identified, ensuring the specificity and sensitivity of the search. The index terms were combined using the Boolean operators "OR" and" AND". Wild cards represented by an asterisk (*) were used to expand the search further. Methodological quality was assessed using an appraisal tool. Studies were selected based on their quality, which was considered when presenting the results and findings within the discussion of this review. The search for this review took place between March 2022 and March 2023.

**Table 1:** Search terms/keywords

Search keywords	Boolean operator combinations
robotics surgery, robotic-assisted surgery, RAS, assistant, nurse, operating room, theatres, communication	(nurs* OR practitioner* OR assistant*) AND (perioperative OR peri-operative) AND (experience* OR perspective OR attitude* OR view* OR obsers* OR familiar* OR aware* OR practis* AND comm* OR interact* AND (robotic surgery)

**Table 2:** Search terms MeSH headings

Concept	Robotics	Robotic-assisted surgery	Perioperative	Nurse	Communication
MeSH	Robotics, Robotic Surgical Procedures, Remote surgery, Assistive robot, Telerobotic, Exoskeletal device	Robotic surgical procedures, Robotic-assisted surgery/surgeries Robotic surgery/surgeries	Perioperative nursing, Surgical nursing Operating rooms	Nurse, Personnel, Nursing Nurse, Registered	Communication
Keywords	Robot surgical robotics	Robotic-assisted surgery	Perioperative, operating room	Nurse registered nurse	Communication

### Inclusion and exclusion criteria

Aveyard, Payne, and Preston [7] recommended adopting a pragmatic approach when limiting publication dates with reasonable justification. The rationale for defining the time frame from 2000 until 2023 would enable the authors to access sufficient papers on a subject with a limited evidence base. Inclusion and exclusion criteria were guided by the literature review aims and in keeping with PICO (Table 3). Abstracts were screened for eligibility guided by the inclusion criteria.

**Table 3:** Inclusion and exclusion criteria

Inclusion criteria	Exclusion criteria
Empirical published articles between January 2000 until March 2023	Research that studied non-surgical robotics
Research included participants such as nurses or theatre practitioners working in the operating theatre/operating room in robotic surgery	Research that did not directly report the experience of the participants under study during robotic surgery
Peer-reviewed published papers	Research on surgical robotics based on laboratory experiments
Quantitative or qualitative, or mixed methods research that directly reported the perception or experience or views of the participants under study	
Published in English language	
Studies from all countries that are meeting the above criteria	
Hospital setting simulation robotic surgery	
Robotics simulation training setting involving nurses	

### Search results

**Fig. 2:**
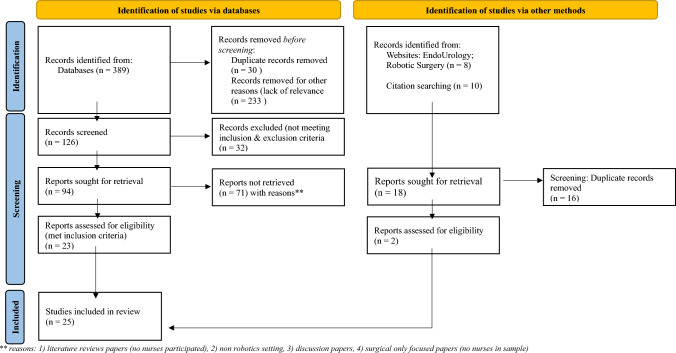
PRISMA diagram. **reasons: 1) literature reviews papers (no nurses participated), 2) non robotics setting, 3) discussion papers, and 4) surgical only focussed papers (no nurses in sample)

The PRISMA diagram (Fig. 2) outlines the screening process and result for the review. Initial 94 papers were retrieved with screening using the inclusion and exclusion criteria, and the primary author reviewed the titles and abstracts. 71 papers were removed with reasons as recorded in double asterisks (**). Following further manual searches from relevant journals and manual citation cross-checks, additional two papers were included. A total of 25 papers published between 2004 and 2021 were included in this review. Kable, Pitch and Masslin-Prothero's [24] framework was adopted to provide the overall data extraction and the findings from the included papers.

## Results

### Study characteristics

Supplementary Table 4 and Table 5 provide a summary of the characteristics of the 25 papers with the respective findings documented. All studies were conducted in the hospital setting. Methodologies used included quantitative studies (*n* = 16) (Table 4), and qualitative (*n* = 9) (Table 5). Eleven countries were represented across the selected studies, with the United States conducting the most research in this area (*n* = 12). The other studies were carried out in Germany (*n* = 3), Turkey (*n* = 1), United Kingdom (*n* = 3), Switzerland (*n* = 1), Korea (*n* = 1), France (*n* = 1), Belgium (*n* = 1), Australia (*n* = 1), and New Zealand (*n* = 1). Thematic analysis (*n* = 4), content analysis (*n* = 2), framework analysis (*n* = 2), and descriptive analysis (*n* = 1) were conducted in the qualitative studies. The sample size varied from 10 to 216, including the number of participants or surgeries observed. While all studies focussed on the impact of RAS on team communication and patient care, including the experiences of the team during robotic implementation, only four empirical papers were authored by registered nurses, with nurses as the primary sample in their investigation [23, 25, 39, 44].

### Methodological appraisal

Supplementary Table 6 and Table 7 provide the quality assessment of all papers. There were no papers excluded from this review during quality appraisal. Studies were chosen for inclusion after the quality appraisal process if they had been published in peer-reviewed journals and if they were based on relevant evidence. The selected studies were based on the relevance of the information provided to the aim of this review. The quality of studies was taken into consideration as presented in the results of this review. Studies were included if they achieved a medium-to-high score on the quality assessment tools. For instance, a study was included when the answer to most of the quality reporting questions was either “Yes” ( +) or “No” ( – ) in CASP quality appraisal tools. The strength of the study rating is based on Low, Moderate, and High as per quality appraisal tools. All studies included a clear description of their design including the methodology, method, recruitment, sampling, data collection, data analysis, and ethical considerations. The 25 papers resulting were quality appraised using Critical Appraisal Skills Programme (CASP) [14]. Appraisal tools guided this review process based on their ability to evaluate each study’s internal validity and trustworthiness. Qualitative papers (*n* = 9) were evaluated using the CASP qualitative assessment tool (2018) and scored separately from the Quantitative papers (*n* = 16) using the RCT assessment tool (2022). A systematic, structured approach was adopted to examine each study's strengths and limitations to help determine its weight and relevance to address the review question. Although some studies reported small sample sizes as their limitations, all research designs met the aims of the studies.

### Data analysis

**Fig. 3:**
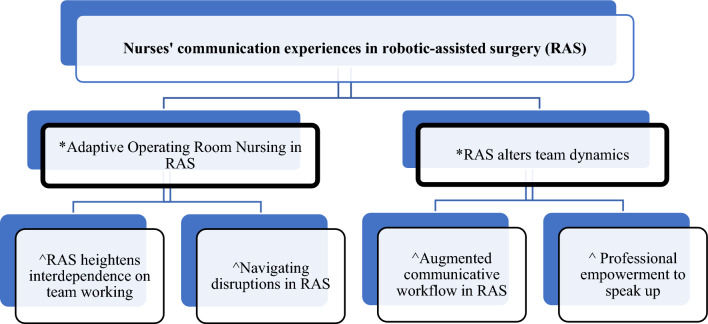
Thematic map *Themes & ^subthemes

Whittemore and Knafl's [48] five stages guided the data analysis through data reduction, presentation, and comparison. The outcomes from the 25 papers were categorised into themes based on common characteristics. Supplementary Fig. 5 detailed the constant comparison of the variables from categories and facilitated a distinction pattern from the data in stage 4, which subsequently developed into themes and subthemes. The thematic map (Fig. 3) provides the visualisation of links and their relationships generated from the categories. The original texts were verified for accuracy and conformity, as Whittemore and Knafl [48] suggested, to ensure the conclusions' validity and minimise interpretation bias and error.

## Results

This review found the quantitative research design (*n* = 16) was the dominant approach to exploring this topic. All studies reported that team communication was key to successful surgical outcomes and patient safety in RAS. Despite the importance of having systems in place to achieve efficiency and safety [10, 23, 25, 30, 45], the review found that the team relationships between the surgeon, nurses, and surgical assistant were highly associated with better communication and interaction in RAS. Randell et al. [35, 36] and Raheem et al. [34] both studies identified team relationships as contributing factors to the successful implementation of a robotics programme.

**Fig. 4:**
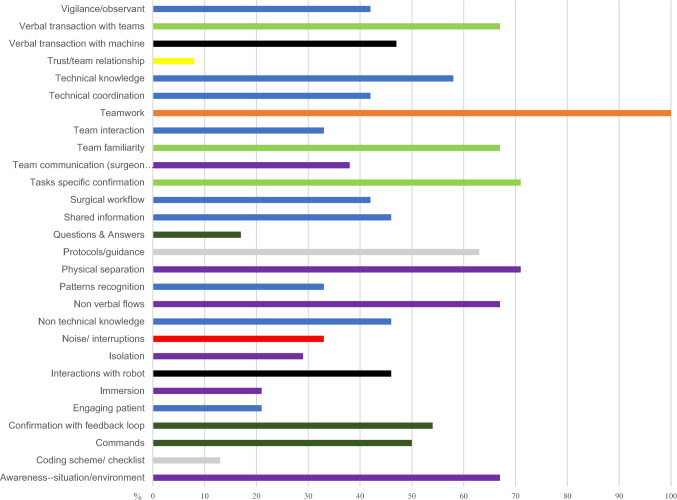
Comparison between the characteristic of communication presented in RAS

**Fig. 5:**
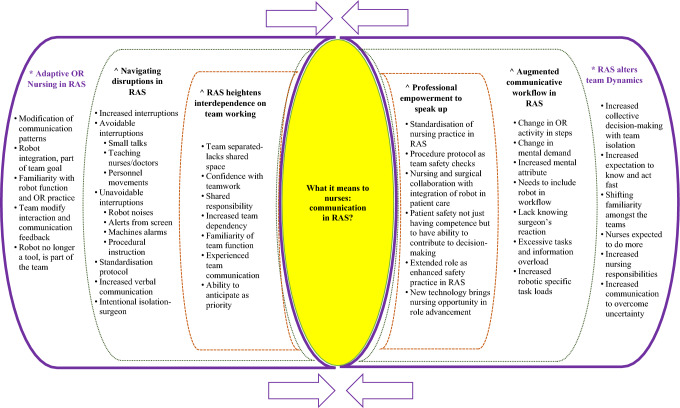
Conceptual model of nurses' communication experiences in RAS

Technologies such as surgical robots bring many advantages to surgical treatment and diagnosis but add another layer of complexity to communication challenges during surgery. Forty-six percent (*n* = 11) of the studies found verbal interaction between the team members on the information from the machines was an additional layer of communication in RAS compared to conventional surgery. The verbal and non-verbal communication reported was notably high, with 67% (*n* = 16) of the studies highlighting these as a form of interaction by the RAS team. In addition, 67% of the studies reported that team familiarity and awareness of the environment when assisting in RAS were associated with surgical efficiency. Physical separation of the team appeared to directly influence the need for more task-specific communication, with 71% (*n* = 17) reported in the included studies. Supplementary Table 8 categorises the numerous characteristics of communication in RAS, which were compared and analysed (Fig. 4) to help develop themes from this review. A total of six themes were generated with a conceptual model, which helps to provide insight for future research (Fig. 5).

Two main themes and four subthemes were developed: ‘*Adaptive operating room nursing in RAS’* with two associated subthemes: ‘*Navigating disruptions in RAS’, and ‘RAS heightens interdependence on team working’*. Secondly: ‘*RAS alters team dynamics’* with two related subthemes: *‘Augmented communicative workflow in RAS*’, and ‘*Professional empowerment to speak up**’*. The findings have presented an understanding of how RAS impacts perioperative nursing practice and team dynamics.

### Adaptive operating room nursing in RAS

The significant reliance of the surgeon on the robotic team is evident from the review. Although communication was the focus during the robotic surgery, there was little emphasis on how it impacts nursing tasks and performance. The nurses reported finding various ways to complete their tasks without impacting patient care outcomes. The shared mental model as a form of communication helps individuals working in the RAS team to know everyone’s role and responsibility and adds to better-informed decision-making and team interaction [10, 13, 23, 26, 34–36, 38, 40].

*Adaptive* is defined in the Oxford Dictionary (2022) as the ability to change to deal with different situations constantly. Cao and Taylor's [10] study shows how nurses encountered an increased cognitive load due to the uncertainty from haphazard communication between themselves and the surgeon. Tiferes et al. [43] reported a new communicative practice using the paired system to communicate accurately between the surgeon and assistant, the surgeon and nurse, or the assistant and scrub nurse. This new approach reflects an original way of getting information seamlessly and effectively compared to other surgeries. In addition, all studies recognised that the nurses and the surgical team had to modify the communication system as an adaptive strategy, such as adjusting their thinking processes and behaviour to cope with the changes in a coordinated fashion.

Role adaptation in practice during RAS varied depending on geographic location, as Cunningham et al. [16] reported. The coordinated way of working and interacting has been previously identified as a central mechanism for safe and effective performance in healthcare and other high-risk work environments [8]. The observation from this review, in particular, the adoption of specific task-based communication, such as during instrument exchanges between the surgeon assistant and scrub nurse, is a significant modification to ensure that safety is observed by the team [10, 43]. In addition, Schiff et al. [38] and Vigo et al. (45) recognised that greater team interdependency demonstrates the nurses’ critical position in RAS. Therefore, as Raheem et al. [34] reported, adopting a standardised communication process would reduce the breakdown of information exchanges. Their study also suggested that verbal communication strongly correlates with efficiency in RAS. Tiferes et al. [42, 43] found that increased reliance on non-verbal interactions, which comes with the introduction of surgical robots, requires team familiarity and a good understanding of the situation. It is, therefore, a false dichotomy only to consider interactions in RAS solely based on verbal and non-verbal acts. Tiferes et al. [43] reported a close association between role and task-specific communication and familiarity with procedures, which impact team interactions. Cavouto et al. [13], Randell et al. [35, 36], Tiferes et al. [43] separately found that this level of understanding helps to reduce the verbal exchanges between the surgeon and the scrub nurse.

Although advances in robotic surgery bring benefits at many levels, they carry unintended complications. Cao and Taylor [10] reported that communication breakdown associated with the complexity of the robotics set-up negatively impacts team function, information flow, and decision-making. Furthermore, Lai and Taylor [26], Nyssen and Blavier [31], Randell et al. [35, 36] all recognised the need for a better understanding of human factors to help integrate the surgical robot to achieve patient safety and quality care. Therefore, these findings revealed increased verbal commands to rectify errors, such as in an emergency, bringing awareness to the entire team as an adaptive operating room practice. In addition, Allers et al. [5], and Almeras and Almeras [4] found that the RAS team, notably the nursing team, used different communication strategies to ensure that the surgeon could hear them. Similarly, as Vigo et al.’s (45) study showed, the surgeon may use a different feedback system to compensate for information missing in the robotic system due to room noise and separation from the team. The following two subthemes will provide a better understanding of why there was a need to navigate disruptions and the heightened interdependency of working as a team in RAS.

#### Navigating disruptions in RAS

Disruptions associated with intraoperative interruptions were reported consistently across this review. It was defined by Catchpole et al. (11, p.3749) as "*deviation from the natural progression of an operation*." There were two forms of disruptions reported: avoidable and unavoidable [4, 5, 16, 17, 27, 42]. In RAS, additional robotics equipment and other patient monitoring machines have an integrated alert system which when activated requires immediate action to be taken by the team as part of the safety measures. El-Hamamsy et al. [17] and Leitsmann et al. [27] found that these unavoidable interruptions from alerts or even machine faults caused distractions to the workflow.

In addition, studies by Cao and Taylor [10], Aller et al. [5], Cavuoto et al. [13], Sexton et al. [40], Almeras and Almeras [4], Tiferes et al*.* [43], and Steffan et al. [41] reported that the new member in the RAS team, whether learning the equipment or the surgery, was considered as an interruption. There are procedural interruptions commonly found in RAS which have increased the workload demand from nurses, such as the need for frequent instrument exchange and attending to surgeons’ requests, including cleaning robotic camera lenses [5, 13, 26, 41, 43, 47]. This observation could be associated with the high quality of the surgeon’s 3D console monitor, which amplifies minute dirt on the lens. An earlier study by Nyssen and Blavier [31] found that reduced interruptions and better coordination resulted from increased team experience in RAS. Therefore, interruptions in RAS significantly disrupt the normal communication flow.

The non-procedure interruptions or risks associated with RAS that were classified as avoidable included noises in the environment from conversations, telephone calls, or physical movements from the OR personnel [4, 5, 16, 17, 27, 42]. The study by Schiff et al. [38] reported an increase in the nurses’ mental load and mental demand due to the high noise level. The complexity of communication with interruptions from multiple perspectives could disrupt the flow of thoughts and information exchanges. Attempts to review the systematic reduction of the noise level in RAS to improve the quality of communication and team interaction, as reported in the study by Leitsmann et al. [27], found no change to the noises related to procedure or others. However, their finding presented a reduction in the noise level from quieter verbal exchanges in the team with special devices worn by the team members. Despite efforts to reduce distractions and improve communication quality in RAS, how they impact the quality of nursing practice in this environment remains unclear.

This review highlighted the concern that preventable disruptions cause unnecessary stress to the surgeon and team, affecting surgical efficiency and potentially jeopardise patient safety. Studies by Almeras and Almeras [4], Cao and Taylor [10], Cunningham et al. [16], Schiff et al. [38], Sexton et al*.* [40], Randell et al. [35], and El-Hamamsy et al. [17] considered the effect of decision-making on safety and the implications of distraction of the surgeon, posed substantial risks to patient safety in RAS. Moreover, Weigi et al. (47) reported that communication demands to overcome surgical flow disruptions in RAS are essential to promote situational awareness to all personnel involved in the surgery. In addition, Jing and Honey [23], Kang et al. [25], Uslu et al. [44], and Schussler et al. [39] recognised the added responsibilities of nurses, including guiding new robotic team members while managing seamless workflow coordination, which adds to their mental burden. Therefore, a fundamental suggestion of how a coordinated robotics team could reduce their mental stress is by adopting a new system of work such as using feedback verbal communication loop on specific task interaction between the nurse, the surgeon assistant, and the surgeon [31, 34, 43]. The specific communication feedback is a form of unique communicative practice from realising surgical robots’ challenges on the surgical team's communication flow.

The surgeon’s immersion in the robotic console, away from the nurses at the bedside, could be a source of reduced interaction with the team. Interestingly, Randell et al. [35] and Almeras and Almeras [4] reported that the surgeon’s isolation has a protective effect of increasing the surgeon’s concentration. However, in the study by El-Hamamsy et al. [17], the concern with surgeon’s isolation in RAS poses communication challenges that impact situational awareness and team emotions. The studies from Aller et al. [5], Randell et al. [35], Weber et al. [46], and Weigi et al. [47] reported no significant associations between disruption and the surgeon’s situational awareness and their perception of stressful demands in RAS. Therefore, the experience of the surgeon’s isolation from the nurses assisting and how it impacts their communication remains unclear in the literature.

El-Hamamsy et al. [17] reported that a dedicated robotic team with a good understanding of their role may need careful consideration to help reduce surgical flow interruptions, thus decreasing perceived stress. In addition, Uslu et al. [44] and Schussler et al. [39] highlighted how nurses' involvement from the start of robotic surgery consistently recognised this factor in contributing to the overall efficiency of RAS. Randell et al. [35, 36] suggested active engagement of the perioperative practitioner, including nurses, to improve the efficiency and implementation of the robotics programme. Moreover, Nyssen and Blavier [31], Kang et al. [25], Aller et al. [5], Sexton et al. [40], and Stefan et al. [41] indicated that having a knowledgeable and skilled workforce is an essential requirement for safe practice in RAS.

This review has shown the challenges nurses face using robotics in practice when the surgical team is in the learning period. Logistical concerns, structural support, and policies were a few of the frameworks that Randell et al. [36] suggested as tools to improve efficiency and maintain patient safety by reducing interruptions in RAS. McCarroll et al. [30], Allers et al. [5], Jing and Honey [23], Kang et al. [25], Uslu et al. [44], and Schuessler et al. [39] also highlighted in their study the impact of extended operating time due to those avoidable interruptions. This contributed to poorer outcomes and lengthened patient recovery. Jing and Honey [23], Kang et al. [25], Uslu et al. [44], and Schuessler et al.’s [39] studies found a significant shift in efficiency with task and information-based communication using checklists to optimise safety in a complex setting. McCarroll et al. [30] and Jing and Honey [23] proposed a pragmatic approach with checklists and protocols to overcome the disruption attached to innovative surgical techniques. Vigo et al. (45) reported that the training protocol identified two important behavioural markers for successful nurse training: eye gaze/contact with the surgeon and anticipating movements to overcome their separation. They have also discussed nursing demands in managing their workloads as required in the operating room. These include technical tasks such as tending to robotic arms and addressing information from the robotic screen with immediate measures to resolve errors.

#### RAS heightens interdependence on team working

The surgeon's physical separation placed a higher reliance on the team during robotics surgery. The studies by Aller et al. [5], Randell et al. [35], Weber et al. [46], and Almeras and Almeras [4] found that surgeon’s immersion in the console significantly impaired their awareness of the environment, which then added to the team members’ burden of responsibility, including nurses assisting at the bedside to ensure safety for the patient while anticipating the surgeon’s needs. Tiferes et al. [43] reported a significant increase in verbal communication (75%) when the surgeon lacked visual evidence from scrub nurses. Therefore, these assumptions may suggest that effective teamwork and cohesiveness from the team are assistive measures to overcome those limitations reported in the previous studies by Allers et al. [5], Cavuoto et al. [13], Weber et al. [46], and Weigi et al. [47].

Furthermore, Cao and Taylor [10], Lai and Entin [26], MaCarroll et al. (50), and Weber et al. [46] stressed the significance of technical coordination among the RAS team to reduce workload and improve flow. Weigi et al. (47) reported the correlation between communication and coordination, which further supports the evidence of increased interdependencies between the workflow processes, the team, and technology. Their findings supported the findings from Uslu et al. [44] and Schuessler et al. [39] that the ability of nurses to actively engage with anticipation improves surgical efficiency and patient safety. These qualities are fundamental to an experienced robotics team to improve care outcomes. Nyssen and Blavier [31] and Tiferes et al. [42] also reported these as a practical solution to the robotics team communication pathway. Moreover, those purposive and task-specific communication pathways taken by the robotics team, including their ability to anticipate the surgeon’s needs, have been identified in studies by Tiferes et al. [42] and Sexton et al. [40] as key variables correlated with team efficiency. The individual's ability to respond quickly and effectively when assisting was found to be helpful in a highly stressful situation, especially to the surgeon. In addition, Almeras and Almeras, [4] pointed out that assistants, including nurses at the bedside, experience a sense of isolation, which adds to their mental load due to increased responsibility as the surgeon is physically separated. Tiferes et al. [42] pointed out that the lack of physical interactions between the bedside team and the surgeon could explain the need for higher verbal communication. A new way of working in RAS presents opportunities to review the team dynamics, the augmented workflow, and the impact of RAS on nursing empowerment to achieve the best outcomes and patient safety.

### RAS alters team dynamics

Robotic surgery has changed the communication dynamics in many ways, as shown in this review. Jing and Honey [23], Kang et al. [25], Uslu et al. [44], and Schussler et al. [39] reported the changes to the nurses’ role and responsibilities that come with the implementation of robotics in surgery. Uslu et al. [44] recognised the changes to assess and support a new way of reviewing the perioperative nurses’ competency by introducing the robot into nursing practice. Moreover, the demand from the nurses in their communication and interaction differs between robotic and non-robotic surgery. The perioperative tasks are no longer simply interactions between two persons. These include intraoperative robotic instrument management, communicating with the surgeon and the team, and knowing how the surgical robot functions and interacts, which play a vital role in intraoperative safety. Both studies by Uslu et al. [44] and Schussler et al. [39] suggest nursing involvement when deciding on any new technology in patient care, such as surgical robots. In addition, the study by Steffen et al. [41] highlights similar findings that support the evolving experience of the perioperative staff. It emphasises the need to carefully consider the robot's impact on their workloads and practice concerns. Undoubtedly, as surgical robot development advances, it comes with a need to review the fundamentals of perioperative practice and robotics safety. In addition to the change in personnel role and function, the robot changes the OR layout with different surgical robotic modalities, which inevitably alters the team dynamics [10, 26, 42]. Furthermore, Randell et al. [36] and Tiferes et al. [43] highlighted the need for technology integration in current and future surgery in any perioperative room design and assessment of surgical patient flow as a strategy to overcome the changes as healthcare technology advances rapidly.

The change in the team members' dynamics, including roles, duties, and task demands, stipulates a higher mental and physical concentration for the nurses [13, 34, 35]. This review reported from studies that the position of the patient and the docking of a heavy robotic cart with arms over the patient are factors contributing to the success of the surgery, but they vary depending on the type of procedure [10, 25, 26, 39, 44]. Nonetheless, Weber et al. [46] reported that the variation in the robotics position and attention to detail needed from the robotics team increases anxiety and stress, especially for the anaesthetist and the bedside nurses. Moreover, Almeras and Almeras [4] emphasised that a safe communication system must be maintained as robotics skills, experience, and behavioural requirements of the team dynamics in RAS. These skills and knowledge come with experience and familiarity among the nurses and the robotic team to achieve efficiency and accuracy. Raheem et al. [34] observed that the alteration of team dynamics is a factor when evaluating patient safety due to incomplete communication. These observations remind the importance of concise task-specific information and the team’s ability to recognise the specific language used with good understanding to overcome team separation as a safety measure. The preference for effective communication from the changes in the team dynamics, either procedural related or associated with the type of robot used, indicates a further understanding of how to support the nurses in the complex environment with evidence to improve communication and their practice.

The scrub nurse is pivotal in any surgical procedure, primarily responsible for instrument management and monitoring patient safety during surgery. However, with the introduction of robots comes a specific change to their practice. The nurses must learn the robotic instruments and their unique features to manage the surgical devices as part of the perioperative practice standards [6]. Contrary to the findings reported by Raheem et al. [34] and El-Hamamsy et al. [17], the scrub nurses were less involved, because the task of the instrument exchange falls under the primary responsibility of the trained surgical assistant. Although Vigo et al. (45) reported the significant challenges demanded from the nurses assisting at the bedside to have active anticipation in RAS, Tiferes et al. [43] reported a virtual space between surgeon and assistant, which could be the explanation for a potential factor that has unintentionally isolated the scrub nurse leading to the perceived lack of active participation in RAS.

It is worth highlighting the multifaceted role of the nurses assisting in RAS to deal with technical challenges, as Vigo et al. (45) reported as one of the indicators for a successful surgical outcome. The studies in Cao and Taylor [10], Cunningham et al. [16], Kang et al. [25], Uslu et al. [44], Schuessler et al. [39], and Steffens et al. [41] recognised that an experienced robotics team is a significant factor contributing to improving team concentration and efficiency, especially during the initial adoption phase, thus creating positive outcomes for the patient. The robot has changed the team in many ways, as described. However, the communication flow is vital in achieving shared understanding in the team and determining the surgical and patient care outcomes. Understanding the task and information flow is an important complex discussion that follows the changes to team and workflow dynamics associated with introducing surgical robots in surgical nursing practice.

#### Augmented communicative workflow in RAS

Current evidence shows that robotic surgery has increased the perioperative nurses' tasks, especially around the management of the robot, including the ability to resolve technical problems when assisting. The robot adds to another level of OR specialist skills demanded from the nurses, as reported by Kang et al. [25], Uslu et al. [44], and Schuessler et al. [39] while not ignoring their mental loads to overcome disruptions from the technology when the robot fails during the surgery. They have to use their technical knowledge to avert any potential patient safety event. Although these novel, unique skills bring specialist advantage to the nurses, they also introduce a different stress level to the nurses and the team when assisting, which they would not encounter in non-robotic surgery. The study by Uslu et al. [44] identified nurses’ fear and hesitation to work with the robot due to a lack of familiarity. Steffen et al. [41] emphasised in their findings to consider the impact of evolving technology on the perioperative staff, suggesting stronger support to the team with safety as the endpoint through better awareness of their attitudes towards implementing robotics in practice. The new pattern of communication amongst the RAS team emerged to compensate for the change, as reported by Aller et al. [5], Weigi et al. (47), Weber et al. [46], and Tiferes et al. [43] helped to understand better about the orientation of workflow and communication associated with the surgical robot technology.

Although task-specific communication flow improves the efficiency in RAS, Sexton et al*.* [41] added that the team's ability to anticipate with better information sharing helps to improve patient safety and OR efficiency from the non-technical perspective. The manner of the information flow in RAS was reported to be significantly augmented to overcome the communication breakdown [16, 31, 34]. In their study, Raheem et al. [34] suggested that standardised communication taxonomy enhances team performance and reduces errors during RAS. Lai and Entin [26], Cavuoto et al. [13], and Weigi et al. (47) reported plausible relationships between the robotic team's uncoordinated task flow and information exchanges, which could suggest a hidden mismatch between task demands, the team’s ability, and the new robotics technology. Therefore, an augmented communicative workflow in RAS helps to overcome adverse events that could be avoidable.

The RAS teams in this review have shown new ways of interaction to convey information and to manage the surgical task effectively and safely. These were all designed to minimise the risk associated with human factors and thus improve patient safety [5, 13, 34, 35, 42, 43]. This review has demonstrated increasingly the robotics team’s awareness of the workflow and better understanding of their collective tasks could foster higher team interdependence. Introducing robots to perioperative nursing practice may seem like an opportunity for the profession rather than a challenge. Understanding the impact of the new ways of work to overcome challenges with the main focus on patient safety will require nurses to have a higher degree of empowerment as surgical robotics continues to play a key role in the surgical patient pathway.

#### Professional empowerment to speak up

Uslu et al. [44], Schuessler et al., [39], and Vigo et al. (45) all show how professional engagement is reflected through empowerment among nurses with better knowledge and skills in robotics. Randell et al. [36] also added that proactive engagement from the nursing profession in robotics influences the success of implementation and care outcomes. The sense of added pressure on the nursing task and their responsibility despite the lack of role clarity when assisting in RAS was emphasised in the studies by Cao and Taylor [10], Lai and Entin [26], Kang et al. [25], and Uslu et al. [44]. However, their studies also show the role of communication effectiveness associated with nurses' empowerment and ability to speak up. The nurses working independently at the bedside with surgeons away at the console can no longer be seen as a concern but rather a reason to empower nurses to speak up confidently. The report from Randell et al. [36] indicated professional appreciation among the perioperative practitioners of the benefits of RAS for the patients but low motivation to participate due to the lack of training opportunities and active involvement during the implementation phase.

Perioperative nursing practice is complex, and the professional confidence to respond effectively entails practice competence and integration of the nursing role in advanced practice such as robotics. Stress and burden to the team were felt by nurses during the transition from learner to expert, especially in the early phase of the robotic programme, when they had to take on extended roles [10, 24, 26, 39, 41, 44]. A supportive environment that empowers nurses to speak up and engage actively helps improve team interaction. However, this demands support for nurses with advanced practice roles and specific robotics protocol development in the practice. Sixteen papers reported the proposal of robotics protocol as guidance for improving workflow efficiency and team training [13, 16, 23, 25, 31, 35, 36, 38–44, 47Vigo et al., 45]. It remains unclear how the protocol would improve nursing engagement from the communication perspective, impacting surgical and patient care outcomes. Despite the lack of clarity in the nursing role during RAS, the study by Uslu et al*.* [44] is the only study that reported the need for process management of robotics surgery as a safety protocol to be established as part of professional legislation to support nursing practice. Despite the recognition to have specialist registration to support nurses' expanding roles and responsibilities from their studies, only Vigo et al. (45) purposively introduced advanced nurse practitioners (ANPs) into their robotics programme as one of their strategies for successful implementation. Undoubtedly, professional autonomy and decision-making were factors associated with the successful adoption of new technology [25, 39, 44], but it requires a more horizontal organisation rather than hierarchical one to reach positive team communication which was found in studies by Uslu et al. [44], Randell et al. [36], and Schussler et al. [39]. Randell et al. [36] asserted in their recommendation that there is still a need for a more collaborative approach to include the perspective of surgical colleagues and managers in implementing any future new robotics system. This includes a significant change in attitude among staff, including nurses who are trained and equipped with a strong foundation of robotics technology [23, 25, 39, 41, 44]. Therefore, nurses' perceived team consistency, better communication, and better care outcomes are associated with greater exposure to robotic surgery and higher empowerment as crucial motivating factors.

## Discussion

This review has synthesised current evidence regarding communication as experienced by registered nurses being part of the robotics team. It reflects limited empirical studies from the nursing perspective to capture the importance of communication in the fast-changing perioperative practice landscape with the exponential introduction of the new generation robotics technology. The strong emphasis on teamwork and team communication across the reviewed evidence forms the foundation for evaluating the nurses' role and communication experiences. The lack of a holistic approach to communication due to the changes in team dynamics and communication flow could be perceived as a barrier to effective team communication and teamwork satisfaction. The team members must coordinate and behave adaptively to compensate for the limitations and challenges faced when assisting in RAS. The characteristics of communication (Fig. 4), which authors^1,2,3^ have reviewed and debated, helped develop the themes as illustrated in Fig. 5. Introducing surgical robots has undoubtedly altered the operating room team dynamics and the physical layout, including the nurses' experiences in communication and team interaction. Interruptions and spatial separation with increased mental demands were a few factors that are closely related when interpreting the relationship of communication in RAS. The implementation of task-specific verbal interaction as a form of adaptive communicative practice would have resulted in a significant alteration in information flow and exchange. Therefore, a better understanding of communication dynamics with multiple characteristics identified from this review is worth noting for future research.

The success of robotics surgery depends on an effective and efficient communication structure and a clear understanding among the team members who are heavily involved in delivering care for the patient under the surgical robot. Navigating surgical disruptions is inevitable with the introduction of robotics in practice but is problematic as it increases the prevalence of surgical incidents. Concerns about mechanical failures, such as instrument or robot faults, could impact the quality of care outcomes [21]. The unavoidable high-level interactions between the nurse and team, considering the physical space of the OR, noises from the environment, the team's mental load, and team members' behaviour, influence new nursing practice in RAS. The introduction of surgical robotics has changed the work structure and perioperative interaction between surgeons and nurses. Instead of simply following the surgeon’s instructions, the nurses assisting in RAS must seek confirmation from the surgeon before completing any specific task. This form of communication adds to safety, unlike the haphazard information exchanges due to lack of familiarity, but it demands that the nurse assisting must have good knowledge and understanding of the surgery and surgical steps. An interesting finding by Randell et al. [36] is that a handpicked dedicated robotic team builds experience, confidence, and efficiency. They cautioned that the team handpicked by the surgeon or nursing management could have a negative impact. Nurses resented being overlooked and, therefore, became disinterested in working with the robot. Undoubtedly, having an expert team familiar with communication and tasks is essential for a successful outcome, but the nursing representation remains under-explored. Team decision-making challenges, the complex nature of RAS affecting teamwork, and errors from the communication breakdown were concerns highlighted consistently in all the studies. Teamwork remains crucial in enhancing surgical efficiency and safety despite advances in robotics.

The OR nurses have added responsibilities such as managing communicative flow during robotics surgery to maintain a state of vigilance. The ability to be actively involved in the safe delivery of care during RAS requires the nurses to have technical and non-technical skills and knowledge, including stronger empowerment to speak up to patient safety. Working in a robotic team with a set-up completely different from the traditional setting adds challenges in the perioperative nursing practice while managing the surgical expectations. The studies by Randell et al. [35] and Raheem et al. [34] stressed the importance of a positive relationship between surgeon and team to foster team confidence. Therefore, understanding the team dynamics and impacts of robotic technology on their interactions and information flow might provide a better understanding of the important cues to determine how to respond to communication concerns experienced by the nurses during robotic surgery. The conceptual model (Fig. 5) synthesised from this review provides a proposed pathway to explore the meaning of communication in RAS from nurses’ perspectives. These findings add clarity to further explore the nurses' function in the complex robotic setting and better understand their perspective of communication that may impact practice and care outcomes.

## Conclusion

Communication in RAS is essential to enhance team performance and patient care outcomes. The two main themes and four subthemes presented in the review have paved the path for discussion when considering the significant function of nurses and the fundamentals of nursing in RAS. Nurses have a core role in perioperative safety and patient care, but limited studies focussing on the nurses' communication experience in this context remain to be explored in future research. This review hopes that the insights into adaptive strategies used to overcome the challenges experienced by the RAS team will allow for a better understanding of the nurses’ communication experiences in future research. Communication in RAS, particularly in OR nursing, requires multifaceted thinking for future practice, education, and research. The review also highlighted the role of organisational leadership and management to include the discussion on multi-level communication and decision-making during the implementation of the robotic programme.

## Relevance to clinical practice

This integrative review has presented new insight into the communication as experienced by the nurses who have a pivotal role in patient safety and surgical outcomes. It provides future direction on how service evaluation should occur when considering the implementation of advanced technology such as robotics. The complexities of advanced technology and nursing practice are emphasised and bring multidimensional challenges to the team in RAS to maintain effective intraoperative communication, team interaction, and familiarity. Understanding these challenges enhances a better perspective of the different groups in the room. Teamwork, communication skills, and the various interactions in the RAS team would add value to practice education, training, and policy implementation for any future introduction of technology in the healthcare system. This review adds to the recognition of checklists as part of the policy to be considered when adopting new technology in the OR to achieve maximum impact on safety practices. Moreover, the findings also suggest careful consideration from the management team or policy holders to take into account of the variation in culture and behaviour of the professionals in a complex operating room setting. Although robotic surgery with better-trained surgeons and teams is considered safe and effective, especially with the benefit to the patient, the evidence reflects a greater awareness is needed to explore strategies to ensure risks associated with communication from the perspective of nurses who have a central role in RAS are assessed and appropriately addressed.

## Limitations

This integrative literature review is part of a doctoral nursing project with an ongoing examination of the evidence on communication-associated impacts in RAS, particularly amongst the nursing workforce. Study selection bias is a risk as this review only included English language only papers and publications between Jan 2000 and March 2023, leaving out any possible newer ones. This review focussed on robotic-assisted surgery only, including non-nursing papers and nurses who were not the key participants as samples in the studies but who were part of the team. These may add to transferability limitations and the risk of reporting bias. Nonetheless, the lack of empirical studies in the areas of interest for this literature review, including team communication, which nurses were part of, will provide a layer of insight for future research.

## Supplementary Information

Below is the link to the electronic supplementary material.

Supplementary file1 (DOCX 42 KB)

Supplementary file2 (DOCX 26 KB)

Supplementary file3 (DOCX 27 KB)

Supplementary file4 (DOCX 35 KB)

## Data Availability

The primary research data is being prepared for analysis. Researcher LL, hopes to disseminate and publish the research findings in a near future.
