# Correction to: What do nurses experience in communication when assisting in robotic surgery: an integrative literature review

**DOI:** 10.1007/s11701-024-01942-6

**Published:** 2024-05-28

**Authors:** Lian Lee, Kathleen Greenway, Sue Schutz

**Affiliations:** Oxford Brookes University, Oxford, UK

**Correction to: Journal of Robotic Surgery (2024) 18:50** 10.1007/s11701-024-01830-z

In the original version of this article, the ORCID “0000-0003-4387-6507” for author Lian Lee and ORCID “0000-0003-2686-9313” for author Kathleen Greenway were inadvertently omitted.

