# Supplementary material for: What do nurses experience in communication when assisting in robotic surgery: an integrative literature review
Source: J Robot Surg. 2024 Jan 27;18(1):50. doi: 10.1007/s11701-024-01830-z (PMC10822005; doi:10.1007/s11701-024-01830-z)
Supplement: Supplementary file 1 — Supplementary file1 (DOCX 42 KB) [file 11701_2024_1830_MOESM1_ESM.docx]

Table 4: Main articles (Quantitative study, n=16) characteristics and summary

| **Author** | **Research Title** | **Research Aims/**  **purpose** | **Context/**  **setting/**  **sample** | **Design** | **Outcome measures / Data generation/** | **Data analysis** | **Results/ Findings (summary)** |
| --- | --- | --- | --- | --- | --- | --- | --- |
| Allers, J. C. *et al*. (2016) | Evaluation and Impact of Workflow Interruptions During Robot-Assisted Surgery | To analyse and categorise causes for interruptions during robot-assisted surgery | Participants:  Urologist; Surgical fellow; Physician Assistant  Country:  USA | Quantitative descriptive study | Observation of recorded videos: Retrospective analysis of 10 recorded robot-assisted prostatectomies  From October 2014-June 2015 | Descriptive statistics—  Means and standard deviations (SD)  Wilcoxon rank-sum test for ordinal data  Pearson Chi-square test for categorical variables  Spearman correlation to test association between interruptions  Cohen’s kappa coefficient to determine inter-rater agreement for qualitative data.  SSAS electronic software was used in all statistical analysis | 10 procedures observed—1848hrs operative time—252 interruptions identified ((9% of the total operative time); each procedure was interrupted for 16 mins (25 interruption events, with each lasted of 39 seconds)  Majority of the interruptions during specimen removal (65%), 21% during lymph node dissection.  14% during urethra-vesical anastomosis.  70% interruptions less than 30 seconds; 4% (8 events) lasted longer than 90%.  2 longest events were associated with adverse events.  14% potential avoidable interruptions.  Most events related to equipment/technology issues followed by non-procedural related events and miscommunications/repetitions  Avoidable interruptions +/-46% of overall interruption time; 19% accounted for the presence of new team members in RAS.  No significant correlations between subjective mental workload (measured by NASA-TLX) and interruptions  Significant negative correlation between team familiarity and avoidable interruptions (*P* = .05) |
| Almeras & Almeras (2019) | Operating room communication in robotic surgery: place, modalities and evolution of a system of interaction | To evaluate the feeling and expectation of the different members of the operating room (OR) team in terms of communication during robotic surgery | Operator(O), operator assistant (OA), scrub nurses (SN)  N=69 responses: SN (n=40), OA (n=10),  O (n=19)  Country:  France | Quantitative | Questionnaires (N=130) | Statistical Analysis  Chi^2^ tests | On work condition and training:  P=0.002 significantly reported from O on team consistency required in robotic surgery. No responses from others.  Overall significant findings P=0.022 reported that communication difficulty in robotic surgery compared to other techniques.  P=0.074 reported on environmental interruption such as noise and heat impact on performance and work flow.  P=0.145 considers negative impact as a factor from lack of communication being addressed in training.  Source of communication:  With P=0.0018 significant in the concept of ‘total immersion’ of the surgeon in robotic surgery which has separated from the surgical team at the patient bedside (P=0.045).  External noise from the environment is perceived a difficulty (P=0.019)  Lack of visual feedback was reported by the surgeon (P=0.091)  The loss of non-verbal exchange and environmental encompassing vision which were present during conventional surgery was perceived as challenges in robotic surgery. Although the total immersion and focus of the surgeon increases concentration, it leads to lack of team interaction that may impact communication exchange and workflow.  Interestingly finding includes the surgeon assistants at the patient side experienced complete isolation and solitude.  Therefore, there is an emphasis on training to focus on a system of intercommunication that is necessary verbal and safe to be systematically implemented and reinforced. Hence to be maintained as skills, experience, habits and a requisite of team dynamic. |
| Cao, C. G. L. & Taylor, H (2004) | Effects of New Technology on the Operating Room Team | To examine the changes in performance and communication pattern within the  OR team as a result of the introduction of a new technology | Two procedures observed  Participants:  OR teams included surgeon, nurses and surgeon assistant in the two procedures  Country:  USA | Quantitative | Observational of the recorded images and audios | Descriptive content analysis | This study observed a high dependency of surgeons on team communication.  The OR team relies on verbal exchanges to communicate and coordinate the surgical procedure.  Significant increased reliance on communication between the team members in RAS during adoption of the new technology.  The introduction of a robotic surgical system into the OR changed the flow of information, as well as the point of access to the information and how that information was shared.  Communication patterns including speech and shared mental models were recognised as factors improving interaction and decision-making in RAS. |
| Cavuoto, L. A. *et al.* (2017) | Improving Teamwork : Evaluating Workload of Surgical Team During Robot-assisted Surgery | To investigate the cognitive and physical workload experienced by each operating room team member for different types of Urologic Procedures | 63 procedures  55 OR team members:  3 surgeons  8 trainee assistants  9 scrub nurse  22 anaesthesiologists  2 bedside assistants  11 circulating nurses  Country:  USA | Quantitative | Observation of recorded videos  Survey questionnaires  Likert scale of 0 to 100 measuring mental demand (MD), physical demand (PD), temporal demand (TD), performance effort and frustration. | Statistical analysis using multivariate analysis of the 6 domains of NASA-TLX  Statistical significant was set at α=0.05  ANOVAs  SPSS version 24 was used | Significant team role p=0.001 on all ratings included MD, PD, TD, performance, effort and frustration.  P=0,04 significant effect of surgical duration on MD;  P=0.02 on PD.  PD score was correlated with the clinical outcome of estimated blood loss (r=0.03; p=0.032).  Procedural complexity was not reported as effect on workload  Interesting to note since trainee start of fellowship negatively correlated with the trainee’s perceived workload (r= -0.56; p<.01)  Team experience and familiarity correlate with MD and PD.  OR layout also presents additional cognitive load to the surgeon and OR team members due to change in the dynamics, lack of physical connection, higher communication demands between the team and surgeon.  Bedside assistant scores are associated with the experience of trainee thus trainee’s competence may be perceived as lower workload by the team.  This study also reported workload differs by role, with bedside assistants having higher PD and TD. This reflects the role of bedside assistants needing to fulfil the surgeon’s requests timely to minimise interruptions during RAS. However, scrub nurses are consistently showing lower workload scores among all TLX scales—most scrub nurses were highlighted experienced in this study. |
| Leitsmann, C *et al*. (2021) | The Silent Operation Theatres Optimisation System (SOTOS) to reduce noise pollution during da Vinci robot-assisted laparoscopic radical prostatectomy | To evaluate SOTOS in the setting of RALP | 16 surgeries with SOTOS, 16 without  Participants:  Surgeons, assistants, scrub nurse and circulating nurse  Country:  Germany | Quantitative | Randomised selection of cases between March and June 2017  Questionnaires | Statistical analysis  Wilcoxon rank sum test comparing between groups  t test  Chi-square test | First SOTOS in robotic-assisted surgery  Significant reduction in noise level using SOTOS.  There was a reduction of 3.6dB in noise level (between 3 dB and 3.8dB) – decrease of sound pressure level by 3dB is equal to a halving of sound intensity. (verbal communication=10dB louder than the ambient noise.  There was no change in the external noise from technical equipment, surgical procedures.  The identified reduction in the noise level was from quiet verbal exchange of dialogue between individuals within SOTOS.  Reduced loud speaking=reduce droplet generated during communication thus may have an impact to the operating practice in time of pandemic problems.  There was report of shorter surgical time within SOTOS, which could be related to better communication improved work flow—positive effect of music use within SOTOS  Possible higher concentration from music using SOTOS improve surgical tasks, enhance communication with possible reduction of stress—however it is unpredictable of the measurement of stress.. |
| McCarroll, M. L. *et al*. (2015) | Development and implementation results of an interactive computerised surgical checklist for robotic-assisted gynaecologic surgery | Two folds:  One:  To develop, validate and implement a Robotic Operating Room Computerised Checklist (RORCC) for use in high volume robotic assisted gynaecologic surgery programme.  Two:  To assess whether implementation of the RORCC had an impact on clinical outcomes of hospital utilisation (30 readmissions) and surgery time. | Convenient sampling  n=4 expert Gyn surgeons  n=32 Operating Room nurses  Country:  USA | Quantitative | Statistical analysis | Content analysis  Prospective review process and retrospective to capture clinical outcomes  Content validity  -expert opinions on checklist items  Face validity—assessed by asking experts to review draft version of checklist  Field testing—during simulation demonstration of checklist | Significant reduction in readmission using checklist, p=0.02  The integration of electronic checklists for Gyn robotic-assisted surgery reduced hospital readmission at the 30 day period without significantly impacting operating room time. Therefore the introduction of RORCC contributes to improved patient safety and decreased health costs |
| Nyssen, A. S. & Blavier, A (2010) | Integrating Collective Work Aspects in the Design Process: An Analysis Case Study of the Robotic Surgery Using Communication as a Sign of Fundamental Change | To study how robotic surgery induces fundamental changes in the collective work using communication as a sign of adaptation processes | Three stages:  First:  Comparing laparoscopic (>100) and robotics (>10): observation  Second:  Compared team with different level of expertise during robotic gynaecology (surgeons and assistants)  Third:  Compared routine and non routine surgeries  Participants: Surgeon,  Surgeon assistant, Nurse  Country: Belgium | Quantitative | Experimental Study  Observations | The Mann-Whitney *U* test was used to compare robotic and laparoscopic techniques  Kruskal-Wallies test was used across all the observations.  Content analysis of communication | Robotic surgery creates a new pattern of communication, p<0.05 significant referring to surgical orientation, manipulation, order and confirmation of the system impact on breakdown between surgeon and assistant.  When there was increased experiences both surgeon and assistant, communication breakdown reduced—p<0.05  This study results show:  Robotic system changes the feedback loop and verbal communication used by surgeons.  Feedback adaptive process was used to compensate for face to face information exchange.  The technical aspect of robotics such as manipulation, orientation and strategies can be learned with interaction with the technical system.  The experience of the teams remains an important confirmation of communication.  Increased verbal communication is required in robotics to enable coordination especially during unexpected events. |
| Raheem, S. *et al*. (2018) | Variability and interpretation of communication taxonomy during robot-assisted surgery: do we all speak the same language | To investigate and analyse the different ways surgeons communicate with bedside assistants during robotic-assisted surgery | 26 procedures; 3 surgeons, 3 physician assistants, 3 scrub nurses  Country:  USA | Quantitative | Observation of five tasks specific in robotic-assisted surgery. (retrospective review audio records)  Comparative of requests and frequency with acknowledgments | Descriptive analysis, comparison using Wilcoxon rank-sum and chi-square rests | five tasks observed, with 166 requests (17%)-associated with inconveniences- (n=81, 33% were clipping related with significant of p=0.88 being acknowledged); instrument change-most frequent communication (P<0.001) trended towards shorter time to complete (P=0.07). suction being the most specific task made ()<0.001) with lowest level of inconvenience, no differ in action time. retraction has similar action time to others, specific retraction requests were acknowledged significantly more often by assistants (P=0.02). significant action time between complete and incomplete requests (p<0.001).  This study provided initial understanding of communication process in RAS, and demonstrated that non-technical skills can be effectively and objectively quantified and measured |
| Schiff, L. *et al*. (2016) | Quality of communication in robotic surgery and surgical outcome | To evaluate the association between the quality of communication and surgical outcomes | Surgeon, surgical nurse and surgical technicians in robotic gynaecology  (N=40 cases; 32 participants)  From March 1 to May 31 2013  Country: USA | Quantitative | Prospective questionnaire-based study  Survey | Statistical analysis  Spearman correlation between individual quality of communication survey items and surgical outcomes  Factors negatively affected the quality of communication:  Noise level in the OR (78%); surgeon console microphone to bedside assistant communication (64%); lack of familiarity of the team (61%). | This study has illustrated the complexity of communication among the robotic team members in a procedure.  Workflow and RAS was mentioned  The non-verbal which traditionally adopted by surgeons during a case have been removed with barriers during RAS from robotic equipment and spatial distance from the sterile field and team. Thus impacting on decision making and information flow that may affect the team function.  The increase level in noise and disruption of workflow during RAS are attributable to increase mental load to the OR team and nurses and affect surgical outcomes |
| Sexton, K., *et al*. (2018) | Anticipation, teamwork and cognitive load: chasing efficiency during robot-assisted surgery (RAS) | To investigate the impact of anticipation as a measure of efficiency in the setting of RAS | 12 surgeries participants:  3 Lead surgeon  3 Assistant surgeon  7 Scrub nurses  11 Circulating nurses  Country: USA | Quantitative | 12 robotic assisted radical prostatectomies—Observational  Analyse video and audio | Descriptive statistical analysis  National Aeronautics and Space Administration—Task Load Index (NASA-TLX) was used to quantify cognitive load  Self-assessment (questionnaires): included six variables—mental demand/ physical demand/temporal demand/perceived performance, effort and frustration were measured from the responses’ score | 1330 requests documented/ 413 (31%) were anticipated  8% reduction on operative time.  Teamwork, communication and familiarity are factors associated with team performance and improve clinical outcomes in surgery.  The impact of the variables measurement were correlated to team efficiency.  Anticipation has been found to be related to team efficiency and their ability to respond to high stress situations.  Anticipation in RAS is measurable and quantified with the use of anticipation ratio.  Surgical experience and team familiarity improve surgical efficiency and patient safety.  This study has highlighted the importance of non-technical factors in improving OR efficiency and ultimately patient safety. |
| Steffens D, *et al*. (2020) | Evolving experience of operating theatre staff with the implementation of robotic assisted surgery (RAS) in the public sector | To examine the knowledge and attitudes of theatre staff before and after implementation of RAS in the public sector | 250 theatre staff recruited with 200 completed the survey: including nursing, medical and support staff.  Country:  Australia | Quantitative:  Longitudinal study  1 week before and 32 months after the implementation of the RAS program) | Survey:  Using three points Likert scale on four items in the questionnaires.  In addition respondents' sex, age category, staff specialty, knowledge and skills level in robotic surgery | Research Electronic Data Capture (REDCap) database was used to store the collected data;  Statistical analyses were performed using IBM SPSS Statistics version 24 (IBM Corp., Armonk, NY, USA);  Chi-squared tests was used to compare the results;  Statistical significance for all analyses was defined as two-tailed  P, 0.05 | Of the total 250 participants, 164 (65.6%; 77, 47 and 40 nursing, medical and 40 support staff respectively) completed the study survey before implementation of the RAS program, and 200 (80.0%; 81, 97 and 22 nursing, medical and 40 support staff respectively) completed the survey after RAS implementation.  Samples characteristics:  No differences in sex, age, robotic knowledge and robotic skills were found within medical and support staff groups before and after the implementation of RAS. With time, nursing staff increased their robotic knowledge (P ¼ 0.009) and robotic skills (P ¼ 0.001).  Knowledge and attitude towards RAS changed after implementation among nursing staff:  They believed RAS benefits the patients with P 0.016 believed it reduces patient's intra-op pain and reduced intra-op complication (P=0.002).  However there was still uncertainly of the benefit of RAS to theatre staff.  Concerns still present among the nursing staff with increased workloads and cost associated with robotic surgery and technology.  Similarly with medical staff who also suggested the benefit of RAS reducing intra-op complication (P=0.003)  There was significant improvement in supporting staff group attitudes towards RAS workplace safety and concern on handling of the equipment (P=<0.001)  Both medical and nursing staff attitudes towards facilitators remained unchanged after implementation, with most agreeing that theoretical, practical training, educational guides and staff support would facilitate the implementation of new technology (SD >88.9%).  Participants' concerns regarding the impact on workplace safety were significantly reduced. |
| Tiferes, J. *et al*. (2016) | The Loud Surgeon Behind the Console: Understanding Team Activities During Robot-Assisted Surgery | To design a methodology to allow comprehensive analysis of team activity during RAS | 37 patients  89 Operating Room staff  Participants: Surgeons, Physician Assistants, Nurses  Country : USA | Quantitative  Field study | Observations-Recorded video analysed via a movie editing software (Noldus Observer XT 12)  Video observations by Human Factors PhD graduate student and 2 trained observers.  Survey questionnaire NASA-TLX was used to ask the team how long they have known each other and worked together. | Descriptive content analysis using electronic video editing software | This study was able to provide characterise team communication in terms of flow, mode, topic and form.  The study also has informed surgical flow being evaluated in terms of duration, location, personnel involved, purpose and if movements were avoidable.  Procedural interruptions were characterised according to their duration, cause, mode of communication and personnel involved.  This study has provided methodology feasibility to capture a wide variety of team activities during RAS which would serve as a solid platform to improve non-technical aspects of RAS.  This study provides characteristic pathways to track team members' interactions during RAS, their purposes and needs. Capturing team movements from this study also provided data for better design of future OR to help improve safety and time efficiency. |
| Tiferes, J. *et al*. (2019) | Are gestures worth a thousand words? Verbal and nonverbal communication during robot-assisted surgery | As part of the ‘Techno Fields’ project to study and improve teamwork, communication and other non surgical skills in RAS  Characterised team verbal and nonverbal interactions among the team members in robot-assisted surgery (RAS) to increase knowledge of how surgical teams communicate during RAS. | Purposive samples—OR teams worked in the 11 recorded robot-assisted radical prostatectomies  (6 surgeons, 2 assistants, 7 scrub/circulating nurses, anaesthetists)  Single centre study  Country: USA | Quantitative | Retrospective observation of audio video recordings | Statistical  Descriptive analysis  Chi-Square analysis of the communication variables occurred in the team interactions during RAS | This study has recorded different teams using both verbal and nonverbal communication strategies in RAS.  There were different communication topics between the roles which was a true reflection of role function and familiarity of the procedures.  The percentage of interactions also reflected the key contribution of role in RAS:  Assistants with 67% being of the strategic position placed in surgery ensuring the surgeon at the console is aware of the events. 26% was with the scrub nurse which again is also a reflection of the experience of the scrub nurse which may vary for new scrub nurses with lack familiarity.  Interesting to read that only 7% interactions between surgeon and scrub nurse. The only communication scrub nurses have with surgeons were on instrument exchanges and camera adjustment.  There was a virtual space which the surgeon and assistant were sharing but not with the scrub nurse. This virtual space reduced the need for constant verbal interactions between them.  Interesting to note that the increase of 75% verbal exchanges between surgeon and scrub nurses were due to little visual evidence of scrub nurse activities to the surgeon especially during removing and installing of instruments.  Therefore the shared view is an important characteristic associated with good team interactions in RAS.  It is a false dichotomy merely considering interactions on communication acts both in verbal and nonverbal.  59% of the robotic team interactions were nonverbal, and were mediated by technology or face to face, OR design including layout.  This study has validated the proportion of verbal and nonverbal communication that seems to be affected by role and the topic of communication. |
| Vigo, F. *et al.* (2021) | An interdisciplinary team training protocol for robotic gynaecologic surgery improves operating time and costs: analysis of a 4 year experience in a university hospital setting | To assess the effectiveness of a structured, interdisciplinary, surgical, team training protocol in robotic gynaecologic surgery, with gradual integration of an advanced nurse practitioner | 175 robotic laparoscopic procedures  Participants: Surgeon, Surgeon Assistant, Advanced Nurse Practitioner  Country : Switzerland | Quantitative | Descriptive study  Testing of training protocol in 4 phases | Statistical descriptive analysis | Importance of continuous training and frequent surgical activity in the operating room.  This paper has presented the similar challenges OR nurses encounter to the medical trainees—nurses are confronted with conceptual and technical challenges.  Two behavioural markers for successful nursing in robotics namely eyes gaze/contact with the surgeon and anticipation movements—which are compromised due to surgeon is sitting away from the sterile field; high technical competence in robotics and the demand of active role from nurses in RAS are multifaceted contributing to successful surgical outcomes.  Training will enhance nurses confidence and quality to overcome individual fear and hesitation when dealing with the robotic system. |
| Weber, J. *et al.* (2018) | Effect of Flow Disruptions on Mental Workload and Surgical Performance in Robotic-Assisted Surgery | To identify flow disruptions and assess their association with mental workload and performance during robotic-assisted surgery | All OR professionals who were present in the OR at least 1 hr and actively involved in the procedure:  Surgeon, surgeon assistant, nurses included scrub nurses and circulating nurses and anaesthetists  93 nurses, 81 surgeons, 42 anaesthetists  Country: Germany | Quantitative  Multi-methods design | Structured observations of robotic surgeries  Operating Room professionals' self-reports survey | The differences in disruptions were calculated with ANOVA statistical measure, using Greenhouse-Geisser p-value in reporting  Correlation analysis was used to determine association between disruption rate and severity of the and OR professionals’ reports  Multiple testing using Bonferroni correction. R 3.3.1 was used for all analysis | Significant interference during robotic-docking p<0.001  Highest severity was reported during instrument exchange.  Anaesthetists reported highest mental workload and performance (p<.001); nurses p=.011  Anaesthetists reported higher intraoperative distractions than surgeons p<.001.  Intraoperative performance rate between professions with highest values reported for surgeons and nurses p<.001 compared to anaesthetists.  Study suggests improved communication and team coordination reduce workload and increase intraoperative performance.  Interestingly this study found low distraction for surgeon immersion in the console and found no significant association between flow disruptions and surgeon’s mental demands, situational stress or performance. The authors suggest the robotic system creates protective effects against disruptive events! |
| Weigi, M. *et al*. (2017) | Associations of Intraoperative flow disruption and operating room teamwork during robotic-assisted radical prostatectomy | To identify type and severity of surgical flow disruption and to determine their impact on the perception of intraoperative team | Operating Room team included surgeon assistant, scrub nurses, nursing trainees and anaesthetists  Country: Germany | Quantitative descriptive study | Multimethod observational study—expert-based observations during surgery and post op participants’ survey  Observations of the events during surgery impacted on workflows by two expert based trainees.  Intraoperative teamwork effectiveness was assessed with participants surveyed post-operatively. | Using linear regression statistical analysis  Unit of analysis: observed procedure and associated OR team’s evaluations | Phase 1 observation (pre-robot phase):  28.2% disruption was communication (most prevalence)  Phase 2 docking of robot:  32% disruption was docking procedure related.  Phase 3 console time:  22.3% Communication related occurred frequently.  External disruptions were accounted for 29% during phase 1, 23% during phase 2 and 30% during phase 3  The team work scored higher by the surgeon compared to nurses and anaesthetist: P<.01  There was close correlation of rating between the surgeon and the nurses but not with the anaesthetist. However interestingly the correlation of rating from nurses were associated with the surgeon and anaesthetist.  The surgical flow disruptions affect perioperative team work.  This study found a significant relationship between communication and coordination during robotic surgery, and provided evidence of interdependencies between process, team and technology in RAS.  This study has highlighted communication demands are essential to promote situational awareness, not just to the surgeon working in console away from the operative field, but to the assistant and nurses.  Robotic instruments exchange and equipment related problems were proportionately reflecting the management of instruments during RAS that has an impact on surgical flow.  Main finding:  Surgical flow disruption is correlated to inferior team work. The emphasis on team communication and mutual coordination during RAS is associated with successful surgical outcome with reduction of disruption. |
