# Supplementary material for: What do nurses experience in communication when assisting in robotic surgery: an integrative literature review
Source: J Robot Surg. 2024 Jan 27;18(1):50. doi: 10.1007/s11701-024-01830-z (PMC10822005; doi:10.1007/s11701-024-01830-z)
Supplement: Supplementary file 2 — Supplementary file2 (DOCX 26 KB) [file 11701_2024_1830_MOESM2_ESM.docx]

Table 5: Main articles (Qualitative study, n=9) characteristics and summary

| Author(s) | Research Title | Research Aims/  purpose | Context/  setting/  sample | Design | Outcome measures / Data generation/ | Data analysis | Results/ Findings (summary) |
| --- | --- | --- | --- | --- | --- | --- | --- |
| Cunningham, S. *et al*. (2013) | Effect of Experience and Workplace Culture in Human-Robot Team Interaction in Robotic Surgery: A Case Study | To present a methodology for examining human-robot team interaction in complex environment | Field study two hospitals in two different countries  Participants:  Teams made up of surgeon, surgeon assistance, scrub nurse and circulating nurse  Country:  France & USA | Qualitative  Field study | Observation of live surgery-  Total of 14.5hr observed in both settings  Semi structured interviews | Content analysis from the interviews and observations of videos  Workflow analysis  Timeline analysis  Communication analysis in each phases of the procedures  Communication pattern analysis  Transcribed verbatim using communication taxonomy to define different verbal exchange topics:  Equipment related  Procedure related  All other topics | Difficult to define human robot interaction in complex and dynamic environments such as OR.  Having examined the phases of the workflows and communications, this study identified the differences of the different phases in robotics impacts by experience levels in the use of robotic systems, its organisation and cultural influences.  Communication analysis highlighted the challenges of adopting new technology in an environment already complex such as OR.  Team behaviour and workplace factors were included as contributing source to the team experiences.  OR interactions and communication patterns (verbal) can significantly affect surgical workflow in RAS.  This study findings also suggest team benefits from common goal training and communication protocols for effective remote collaboration.  Summary from this study demonstrates an effective methodology that is applicable to study human-robot team interaction in a dynamic work environment. |
| El-Hamamsy, D. *et al*. (2020) | Surgeon-Team Separation in Robotic Theatres: A Qualitative Observational and Interview Study | To examine in depth how separation in robotic theatres affect team dynamics and staff emotions | Participants:  Consultant Surgeon, Trainee Doctor,  Surgeon Assistant  Anaesthetists, scrub nurse, ODP,  Healthcare Assistant/  Care Support Worker  Country:  United Kingdom | Qualitative | Observation of robotic procedures  (N=109) in two tertiary hospitals, compared with laparoscopic/open surgeries—field notes  Participants observed: N=29  One to one interviews (N=26), audio recorded and transcribed verbatim | Qualitative analysis using Grounded Theory approach with NVIVO11 | Three themes reported:  Communication challenge  Immersion vs distraction  Emotional impact  Additional compounding factors included:  Individual and team experience, staffing levels and the physical environment of the robotic theatres |
| Jing, J. M., & Honey, M. L. (2016) | Using a Checklist in Robotic-Assisted Laparoscopic Radical Prostatectomy Procedures | To evaluate staff members’ perception of the effect using a checklist whether they believed it improved the performance and safety of the robotic procedures. | Purposive sampling  Participants:  11 staff working in RALRP, who attended robotic training workshop:  Registered Nurse, Physician Assistants  Country:  New Zealand | Qualitative | Focus group  Audio taped | Thematic Analysis  Five stage TA (iterative & reflexive); transcribe verbatim | Four themes:  Confidence in practice  Comprehensive OR set up  Time efficiency  Improved teamwork  This study reported the usefulness of using checklists as a tool of patient safety during robotic surgery with emphasis on a coordinated effort from teams. |
| Kang M. U., Gagne J. C., & Kang H. S. (2016) | Perioperative nurses' work experience with robotic surgery: A focus group study | To explore the work experience of perioperative nurses involved in robotic surgery | 15 surgical robotic nurses:  Participants:  One male and 14 female nurses (mean age, 31.33 [SD, 4.19] years; range, 25–41 years). Their experience as robotic surgery nurses ranged from 8 months to 6 years.  Country: Korea | Qualitative:  Descriptive study | Focus Group interviews | Qualitative content analysis  No record of thematic analysis however it was briefly reported in the paper:  Credibility was achieved with member checking and peer debriefing, checking for the representativeness of the data  Dependability was achieved by conducting an inquiry audit.  Transferability was ensured by rich description and saturation, and the use of purposive sampling to include participants. | Four themes emerged from findings:  Constant checking on patients' safety and the robot's functions  Unexpected robotic machine errors or malfunctions  Feelings of burden in a robotic surgical team  Need and desire for more information and education |
| Lai, F. & Entin, E (2005) | Robotic Surgery and Operating Room Team | To identify human factors consideration to address integration of new technologies in the OR | Purposive sampling  24 participants:  Surgeon, Assistant, Anaesthetist, Nurse  13 robotic users, 7 academic researchers, 4 developers | Qualitative | Interviews  Face to face & Telephone | Descriptive analysis | Common themes from this study:  New way of surgery leading to modified roles for the OR team members  New ways of working as OR team with introduction of robots as part of the team which require team coordination in decision-making.  Team members recognised a need for team training with emphasis on predefined protocol in unexpected events during robotic surgery.  Shared situation awareness is key to team members having the same information. |
| Randell R *et al.* (2017) | Impacts of Robotic Surgery on Decision Making: Perspective of Surgical Teams | Part of a process of evaluation, running alongside ROLARR multicentre randomised control trial comparing laparoscopic and robotic surgery for the curative treatment of rectal cancer | 44 operating room personnel  Surgeon (n=12); Surgical trainee (n=5); Manager (n=1); Anaesthetist (n=6); Nurse (n=13); ODP (n=7);  Between Jan to August 2014  Country:  United Kingdom | Qualitative | Face to face interviews | Framework analysis using NVivo 10 | Themes identified were grouped into theories:  Situation awareness; lack of tactile information; immersion; impact of ergonomics  Using a realist approach, this study has suggested experiences teams impact on intraoperative decision-making.  The findings from this study also fit with the author's previous literature reviews. The study has also highlighted that team communication includes the state of the patient and robot. This requires a positive relationship between surgeon and team, which impact the adoption of robotic surgery and team confidence. |
| Randell, R. *et al*. (2019) | Factor supporting and constraining the implementation of robot-assisted surgery: a relist interview study | To answer:  How and in what context does robot-assisted surgery become integrated into practice? | Snowballing  44 staff from 10 Hospitals  Surgeon (n=12); Surgical trainee (n=5); Manager (n=1); Anaesthetist (n=6); Nurse (n=13); ODP (n=7);  Country: United Kingdom | Qualitative  (Part of a process of evaluation, running alongside ROLARR multicentre randomised control trial comparing laparoscopic and robotic surgery for the curative treatment of rectal cancer) | Semi-structured interview (telephone)—mean length 53mins  Audio-recorded; transcribe verbatim | Framework Analysis | The integration of robot-assisted surgery in practice were found to be associated with the following theories from this study:  Dedicated robotic team adds to learning opportunity, increased familiarity thus improved efficiency.  Dedicated operating theatre with appropriate size improved efficiency as it reduces the set up time and end of the case turn around preparation.  Hospital strategies were incorporated into the implementation process  Whole team training enhances relationships, allows the team to resolve problems confidently as a team.  A sense of privilege as being handpicked to be in the robotic team and increased motivation when faced with challenges.  Although lack of team involvement in decision in the introduction of robot-assisted surgery. There was a sense of pride and the experience adds to their professional CV and development.  There was also noted in this study the perceived lack of control over decision in the implementation of robot and also perceived bias from the manner of handpicking staff into the robotic team.  Interesting to read in this report the lack of positive attitude towards robot from nurses and ODPs due to lack of training opportunity. |
| Schuessler Z, Scott Stiles A, & Mancuso, P. (2020) | Perceptions and experiences of perioperative nurses and nurse anaesthetists in robotic‐assisted surgery | To identify the factors that affect nursing care of patients who undergo robotic‐assisted laparoscopic surgery (RALS) | 17 participants: 6 preoperative and postoperative nurses, 7  intraoperative nurses, and 4 CRNAs in the United States  Conducted between 26 April 2014- 24 June 2018  Country: USA | Qualitative:  Descriptive study/naturalistic inquiry | Semi‐structured interview | Thematic Analysis  COREQ checklist to report data collection, analysis and results | This study supports the global clinical community with interventions needed to incorporate in adopting RALS into clinical practice. include standardised  education for the healthcare team, standardised protocols for implementation of the RALS, and measuring outcomes (positive and negative).  The findings demonstrated a need for a universal agreed education programme incorporating simulation modules, laparoscopic including robotic component trouble shooting during emergency—team training and skills training for individual and group.  Emphasises the necessity of the focus on all surgical team members to effectively implement complex technologies, such as RALS. Patient outcomes improved when the RALS team members worked together more efficiently.  Indicates that new technologies may be more effective for some situations, but not necessarily more effective for all situations. Untoward outcomes are part of the implementation of new technologies, and these should be monitored and reported in order to maintain safety for both the healthcare team and their patients.  Decision making from professional nurses during adoption of technology which would impact on their workflows and patient care has been an important criterion in implementation of technology and product evaluation. |
| Uslu Y, Altınbaş Y, Özercan T, van & Giersbergen M. Y. (2019) | The process of nurse adaptation to robotic surgery: A qualitative study | To reveal the experiences of nurses in robotic surgery and their adaptation to this method. | 15 participants: robotic surgery nurses  Country: Turkey | Qualitative: | Semi-structured interview.  Two focus group interviews with 15 participants—first interview was with 8 participants; second interview was with 7 participants.  Each interview lasted 80-120 minutes.  Purposive sampling from four hospitals in Istanbul. | Colaizzi phenomenological interpretive method | Four categories were recorded in the findings:  Training in robotic surgery  Compliance with technology in surgical robotic nursing  Changing role of surgical robotic nursing and adaptive process  The future of robotic surgery  Findings suggested nurses should be experienced in the robotic surgery field.  The competence level of robotic surgery nurses should be determined within the institution and that the duties, authorities, and responsibilities of robotic surgery nurses are not clear, and they are not aware of their competencies.  It is recommended that process management protocols related to robotic surgery nursing be prepared to establish legal bases institutionally or nationally through which the duties, authorities, and responsibilities of the robotic surgery nurses are determined and that internal arrangements be made to improve the working conditions of robotic surgery nurses. |
