# Supplementary material for: What do nurses experience in communication when assisting in robotic surgery: an integrative literature review
Source: J Robot Surg. 2024 Jan 27;18(1):50. doi: 10.1007/s11701-024-01830-z (PMC10822005; doi:10.1007/s11701-024-01830-z)
Supplement: Supplementary file 3 — Supplementary file3 (DOCX 27 KB) [file 11701_2024_1830_MOESM3_ESM.docx]

Table 6: Quality assessment-Qualitative studies

|  | Cunningham et al. (2013) | El-Hamamsy et al. (2020) | Jing and Honey (2016) | Kang et al. (2016) | Lai and Entin (2005) | Randell et al. (2017) | Schuessler et al. (2020) | Uslu et al. (2019) |
| --- | --- | --- | --- | --- | --- | --- | --- | --- |
| Was there a clear statement of the aims of the research? | + | + | + | + | + | + | + | + |
| Is a qualitative methodology appropriate? | + | + | + | + | + | + | + | + |
| Was the research design appropriate to address the aims of the research? | + | + | + | + | + | + | + | + |
| Was the recruitment strategy appropriate to the aims of the research? | + | + | + | + | + | + | + | + |
| Was the data collected in a way that addressed the research issue? | + | + | + | + | + | + | + | + |
| Has the relationship between the researcher and participants been adequately considered? | + | + | + | + | + | + | + | + |
| Have ethical issues been taken into consideration? | + | + | + | + | + | + | + | + |
| Was the data analysis sufficiently rigorous? | + | + | + | + | + | + | + | + |
| Is there a clear statement of findings? | + | + | + | + | + | + | + | + |
| Will the results help locally/ how valuable is the research? | + | + | + | + | + | + | + | + |
| Assessment: High, Moderate, Low | H | H | M | M | M | H | H | H |

Key: + Yes; - No: ct- can't tell; na- not applicable; H-High; M-Moderate; L-Low

Table 7: Quality assessment-Quantitative studies

|  | Allers et al. (2016) | Almeras and Almeras (2019) | Cao and Taylor (2004) | Cavuoto et al. (2017) | Leitsmann et al. (2021) | McCarroll et al. (2015) | Nyssen and Blavier (2010) | Raheem et al. (2018) | Schiff et al. (2016) | Sexton et al. (2018) | Steffen et al. (2020) | Tiferes et al. (2016) | Tiferes et al. (2019) | Vigo et al. (2021) | Weber et al. (2018) | Weigi et al. (2017) |  |
| --- | --- | --- | --- | --- | --- | --- | --- | --- | --- | --- | --- | --- | --- | --- | --- | --- | --- |
| Did the study address a clearly focused issue? | + | + | + | + | + | + | + | + | + | + | + | + | + | + | + | + |  |
|  |  |  |  |  |  |  |  |  |  |  |  |  |  |  |  |  |  |
| Was the cohort recruited in an acceptable way? | + | + | + | + | + | + | + | + | + | + | + | + | + | + | + | + |  |
|  |  |  |  |  |  |  |  |  |  |  |  |  |  |  |  |  |  |
| Was the exposure accurately measured to minimise bias? | na | na | - | + | + | - | - | - | - | - | - | na | ct | na | - | na |  |
|  |  |  |  |  |  |  |  |  |  |  |  |  |  |  |  |  |  |
| Was the outcome accurately measured to minimise bias? | na | na | - | + | + | - | - | - | - | - | - | na | ct | na | - | na |  |
|  |  |  |  |  |  |  |  |  |  |  |  |  |  |  |  |  |  |
| Have the authors identified all important confounding factors? | - | + | - | + | + | - | ct | - | na | na | na | ct | _+_ | + | - | ct |  |
|  |  |  |  |  |  |  |  |  |  |  |  |  |  |  |  |  |  |
| Have they taken account of the confounding factors in the design and/or analysis? | - | + | - | + | + | - | ct | - | na | na | na | na | - | + | - | ct |  |
|  |  |  |  |  |  |  |  |  |  |  |  |  |  |  |  |  |  |
| Was the follow up of subjects complete enough? | - | na | - | na | na | na | na | - | na | na | na | na | na | na | na | na |  |
|  |  |  |  |  |  |  |  |  |  |  |  |  |  |  |  |  |  |
| Was the follow up of subjects long enough? | - | na | - | na | na | na | na | - | na | na | na | na | na | na | na | na |  |
|  |  |  |  |  |  |  |  |  |  |  |  |  |  |  |  |  |  |
| Did the article reports the results of the study? | + | + | + | + | + | + | + | + | + | + | + | + | + | + | + | + |  |
|  |  |  |  |  |  |  |  |  |  |  |  |  |  |  |  |  |  |
| Were the results accurate with clear records of confidence? | + | + | - | + | + | - | + | + | + | + | + | + | + | + | + | + |  |
|  |  |  |  |  |  |  |  |  |  |  |  |  |  |  |  |  |  |
| Do you believe the results? | + | + | + | + | + | + | + | + | + | + | + | + | + | + | + | + |  |
|  |  |  |  |  |  |  |  |  |  |  |  |  |  |  |  |  |  |
| Can the results be applied to the local population? | + | + | + | + | + | + | + | + | + | + | + | + | + | + | + | + |  |
|  |  |  |  |  |  |  |  |  |  |  |  |  |  |  |  |  |  |
| Do the results of this study fit with other available evidence? | + | + | + | + | + | + | + | + | + | + | + | + | + | + | + | + |  |
|  |  |  |  |  |  |  |  |  |  |  |  |  |  |  |  |  |  |
| Was there sufficient report on the implications of study for practice? | + | + | + | + | + | + | + | + | + | + | + | + | + | + | + | + |  |
|  |  |  |  |  |  |  |  |  |  |  |  |  |  |  |  |  |  |
| Assessment: High, Moderate, Low | H | H | M | H | H | M | H | H | H | H | H | H | H | H | H | H |  |
|  |  |  |  |  |  |  |  |  |  |  |  |  |  |  |  |  |  |

Key: + Yes; - No: ct- can't tell; na- not applicable; H-High; M-Moderate; L-Low
