# Supplementary material for: What do nurses experience in communication when assisting in robotic surgery: an integrative literature review
Source: J Robot Surg. 2024 Jan 27;18(1):50. doi: 10.1007/s11701-024-01830-z (PMC10822005; doi:10.1007/s11701-024-01830-z)
Supplement: Supplementary file 4 — Supplementary file4 (DOCX 35 KB) [file 11701_2024_1830_MOESM4_ESM.docx]

Table 8: Categories of communication reported in papers

|  | **Verbal transaction between team** | **Verbal transaction with machine** | **Non verbal flows** | **Noise/ interruptions** | **confirmation with feedback loop** | **tasks specific confirmation** | **teamwork** | **Trust/team relationship** | **team communication (surgeon dependency)** | **Isolation** | **physical separation** | **Technical knowledge** | **Technical coordination** | **non technical knowledge** | **Patterns recognition** | **Team familiarity** | **Awareness--situation/environment** | **commands** | **protocols/guidance** | **Questions & Answers** | **shared information** | **Interactions with robot** | **engaging patient** | **vigilance/observant** | **team interaction** | **surgical workflow** | **immersion** | **coding scheme/ checklist** |
| --- | --- | --- | --- | --- | --- | --- | --- | --- | --- | --- | --- | --- | --- | --- | --- | --- | --- | --- | --- | --- | --- | --- | --- | --- | --- | --- | --- | --- |
| Allers et al., (2016) | 1 | 1 |  |  | 1 | 1 | 1 |  |  | 1 | 1 |  |  |  |  | 1 | 1 | 1 |  |  |  | 1 |  |  | 1 |  | 1 |  |
| Almeras & Almeras (2019) |  |  |  |  |  |  | 1 |  |  | 1 | 1 |  |  |  |  | 1 |  |  |  |  |  |  |  |  |  |  |  |  |
| Cao and Taylor (2004) |  |  |  |  |  |  | 1 |  | 1 |  | 1 |  | 1 |  |  | 1 |  |  |  |  |  |  |  |  |  |  |  | 1 |
| Cunningham S *et al.* (2013) | 1 |  | 1 |  |  | 1 | 1 |  |  | 1 | 1 |  |  |  | 1 |  | 1 | 1 | 1 | 1 | 1 | 1 |  |  | 1 | 1 |  |  |
| El-Hamamsy *et al*. (2020) |  |  | 1 |  |  |  | 1 |  |  | 1 | 1 |  |  |  |  | 1 |  |  |  |  |  |  |  |  |  |  |  |  |
| Jing and Honey (2016) | 1 |  | 1 |  | 1 | 1 | 1 |  |  |  |  | 1 |  | 1 |  | 1 | 1 | 1 | 1 |  | 1 |  |  | 1 | 1 |  |  | 1 |
| Kang MJ *et al*. (2016) | 1 | 1 | 1 |  |  | 1 | 1 |  |  |  |  | 1 | 1 |  |  | 1 |  |  | 1 |  |  | 1 | 1 | 1 |  |  |  | 1 |
| Lai and Entin (2005) |  |  |  |  |  | 1 | 1 |  | 1 |  | 1 | 1 | 1 |  |  |  | 1 |  |  |  | 1 |  |  |  | 1 | 1 |  |  |
| Leitsmann C *et al*. (2021) | 1 |  | 1 | 1 | 1 | 1 | 1 |  | 1 |  | 1 |  |  | 1 |  | 1 | 1 |  |  |  |  |  |  |  |  |  |  |  |
| McCarroll ML *et al*. (2014) |  |  |  |  |  |  | 1 |  | 1 |  | 1 |  | 1 |  |  |  |  |  |  |  |  |  |  |  |  |  |  |  |
| Nyssen AS and Blavier A (2010) | 1 | 1 | 1 | 1 | 1 | 1 | 1 |  |  |  |  | 1 |  | 1 | 1 | 1 | 1 | 1 | 1 |  | 1 | 1 | 1 | 1 |  |  |  |  |
| Randell R *et al.* (2017) | 1 | 1 | 1 |  |  |  | 1 | 1 |  | 1 | 1 |  |  |  |  | 1 | 1 |  | 1 |  | 1 |  |  | 1 |  |  | 1 |  |
| Raheem S *et al.* (2018) | 1 |  | 1 |  | 1 | 1 | 1 | 1 | 1 | 1 | 1 | 1 | 1 | 1 | 1 |  | 1 | 1 |  |  |  |  |  |  | 1 | 1 |  |  |
| Randell *et al*. (2019) |  |  |  |  |  |  | 1 |  |  |  |  |  |  |  |  | 1 |  |  | 1 |  |  |  |  |  | 1 |  |  |  |
| Schiff L *et al.* (2016) | 1 | 1 |  | 1 | 1 | 1 | 1 |  |  |  |  | 1 |  |  | 1 |  | 1 | 1 | 1 |  | 1 | 1 |  |  |  |  |  |  |
| Schussler Z *et al*. 2020 | 1 | 1 | 1 |  |  | 1 | 1 |  |  |  |  | 1 |  | 1 | 1 |  | 1 |  | 1 |  | 1 | 1 | 1 | 1 |  |  |  |  |
| Sexton K *et al*. (2018) | 1 | 1 |  | 1 | 1 | 1 | 1 |  |  |  |  |  |  |  | 1 | 1 |  | 1 | 1 |  |  | 1 |  |  |  |  |  |  |
| Steffens D *et al*. (2020) |  |  |  |  |  |  | 1 |  |  |  | 1 | 1 |  | 1 |  | 1 |  |  | 1 |  |  |  | 1 |  |  | 1 |  |  |
| Tiferes J *et al*. (2016) | 1 | 1 | 1 | 1 |  |  | 1 |  |  | 1 | 1 |  | 1 |  | 1 | 1 |  | 1 | 1 | 1 |  | 1 |  |  |  | 1 |  |  |
| Tiferes J *et al.* (2019) | 1 | 1 | 1 | 1 | 1 | 1 | 1 |  |  |  | 1 | 1 | 1 | 1 | 1 |  | 1 | 1 | 1 | 1 | 1 | 1 | 1 | 1 |  | 1 |  |  |
| Uslu Y *et al*. (2019) | 1 | 1 | 1 |  | 1 | 1 | 1 |  |  |  |  | 1 |  | 1 |  | 1 | 1 | 1 | 1 | 1 |  | 1 |  | 1 | 1 |  |  |  |
| Vigo F *et al*. (2021) | x |  | 1 |  | 1 | 1 | 1 |  | 1 |  | 1 | 1 | 1 | 1 |  | 1 | 1 |  | 1 |  | 1 |  |  | 1 | 1 | 1 |  |  |
| Weber J *et al.* (2018) | x | 1 | 1 | 1 | 1 | 1 | 1 |  | 1 |  | 1 | 1 | 1 |  |  | 1 | 1 | 1 |  |  | 1 | 1 |  | 1 | 1 | 1 | 1 |  |
| Weigi M *et al*. (2018) | 1 |  | 1 |  | 1 | 1 | 1 |  | 1 |  | 1 | 1 |  | 1 |  |  | 1 |  | 1 |  |  |  |  |  |  | 1 | 1 |  |
| **counts** | **16** | **11** | **16** | **8** | **13** | **17** | **25** | **2** | **9** | **7** | **17** | **14** | **10** | **11** | **8** | **18** | **16** | **12** | **17** | **4** | **11** | **11** | **5** | **10** | **10** | **10** | **5** | **3** |
| **% (percentage of counts)** | **67** | **46** | **67** | **33** | **54** | **71** | **100** | **8** | **38** | **29** | **71** | **58** | **42** | **46** | **33** | **68** | **67** | **50** | **65** | **17** | **46** | **46** | **21** | **42** | **36** | **42** | **21** | **13** |
